# Slow virologic control but strong immune and metabolic recovery with dolutegravir-anchored therapy in an HIV cohort in Ghana

**DOI:** 10.1186/s12985-025-02873-w

**Published:** 2025-07-19

**Authors:** Mark Appeaning, Edwin Magomere, Alberta Mawulawoe Abotsi, Nana Ama Yeboaa Amoako, Kirk Elorm Kouffie, Becky Ewurama Tetteh, Bridget Nana Darkoa Quist, Christèle Nguepou Tchopba, Gloria Akosua Ansa, Evelyn Yayra Bonney, Peter Kojo Quashie

**Affiliations:** 1 West African Centre for Cell Biology of Infectious Pathogens (WACCBIP), College of Basic and Applied Sciences, University of Ghana, P.O. Box LG54, Legon, Accra 23321 Ghana; 2 Department of Biochemistry Cell and Molecular Biology, School of Biological Sciences, College of Basic and Applied Sciences, University of Ghana, P.O. Box LG54, Legon, Accra 23321 Ghana; 3 Department of Medical Laboratory Science, Faculty of Health and Allied Sciences, Koforidua Technical University, P.O. Box KF981, Koforidua, 03420 Ghana; 4 Public Health Department, University of Ghana Health Services, Accra, Ghana; 5 Noguchi Memorial Institute for Medical Research, University of Ghana, P.O. Box LG 581, Accra, Ghana; 6 The Francis Crick Institute, 1 Midland Road, London, UK

**Keywords:** WHICH study, HIV cohort, HIV cure research, West Africa, Ghana, HIV-1, HIV-2, HIV 1 & 2 dual infection

## Abstract

**Introduction:**

The West African HIV/AIDS epidemic, historically driven by HIV-1 CRF02_AG, other recombinant forms and HIV-2, remains less researched for various preventive and therapeutic interventions. We established the **W**ACCBIP long-term **H**IV **I**nfection **C**o**h**ort (WHICH Study) to investigate the dynamics of HIV epidemic in Ghana. This report evaluates viral load dynamics, immune responses, and organ-level metabolic changes following antiretroviral therapy (ART) initiation.

**Method:**

We collected blood samples, medical, and demographic data from ART-naïve individuals at baseline and six months post-ART, and from ART-experienced individuals at a single time point. Participants, aged 10 years and above, were purposively enrolled from six health facilities. Laboratory analyses included viral load, CD4 and CD8 counts, co-infection screening (hepatitis B/C, syphilis), liver and kidney function tests, haemoglobin estimation, and HIV-1/2 typing. Chi-square and logistic regression analyses were used to assess associations between participant demographics and clinical data with uncontrolled viremia and immune recovery.

**Results:**

A total of 426 participants were recruited, comprising 159 ART-naïve and 267 ART-experienced individuals, with a mean age of 41.5 years. Median ART duration for ART-experienced was greater than 5 years. Infections included HIV-1 (78.6%), HIV-2 (2.1%), and dual HIV-1&2 (19.2%). Common comorbidities were anaemia (54.9%), hepatitis B (9.5%), and hypertension (8.2%). Most participant (97.9%) were on dolutegravir-anchored regimen. Among ART-naïve individuals, median viral load decreased from log_10_ 5.16 at baseline to log_10_ 4.64 copies/mL after six months (*p* = 0.0156). Median viral load for the ART-experienced arm was log_10_ 3.23 copies/mL. Median CD4 count increased from 290 cells/mm³ in ART-naïve participants to 504 cells/mm³ at six-months post-ART (*p* = 0.0003) and 581 cells/mm³ in ART-experienced participants (*p* < 0.0001). ART-naïve participants were 19 times more likely to have unsuppressed viral loads at baseline compared to ART-experienced participants. ARTnaïve- participants had significantly decreased odds of immune recovery (aOR = 0.35, 95% CI: 0.140–0.85, *p* = 0.021), as did those with low CD4/CD8 ratio (aOR = 0.06, 95% CI: 0.02–0.20; *p* < 0.001). Kidney function and haemoglobin levels were significantly improved six-month post-ART among the ART-naïve group.

**Conclusion:**

This study highlights the significant reduction in viral load and improved immune recovery following ART initiation despite uncontrolled viremia in a subset of participants. This cohort presents an opportunity to study Ghana’s local HIV epidemic, including HIV-1 and HIV-2, and impact of ART on disease progression.

**Supplementary Information:**

The online version contains supplementary material available at 10.1186/s12985-025-02873-w.

## Introduction

To address Human Immunodeficiency Virus (HIV)/Acquired Immunodeficiency Syndrome (AIDS) as an epidemic of public health concern by 2030, eliminating virus transmission in all sub-epidemics across the globe is imperative [1]. A fully suppressed individual does not transmit HIV, and as such, viral suppression is the single most important predictor of progress against this virus [2]. One of the two known types of HIV, HIV-2, originated from West Africa through zoonotic infection from sooty mangabeys and remains largely in this subregion due to its low transmissibility [3]. There is also evidence of continuous zoonotic transmission, circulating recombinant forms (CRFs) and unique recombinant forms (URFs). Unlike the rest of Africa, the West African epidemic is predominantly driven by HIV-1 CRF-02_AG, along with other recombinant forms and HIV-2 [4, 5]. The CRF02_AG accounts for 7.7% of the worldwide pandemic [4], yet this West African epidemic is understudied and has made minimal contributions to drug development, antibody discovery, and cure studies. These subtypes and recombinants reportedly exhibit low pathogenicity and studying them may provide clues to ultimately eliminate HIV transmission and eventually AIDS. Furthermore, CRFs and other non-B subtypes have been associated with nonclassical resistance profiles in dolutegravir-anchored regimens [6].

Cohorts have been essential in understanding local HIV epidemics as well as providing platforms for addressing social issues related to cure strategies. The Females Rising through Education, Support and Health (FRESH) Cohort in South Africa for example has helped in understanding the biological factors that affect the acquisition of HIV among young women as well as the immune response to hyperacute infection [7]. It is imperative to establish a well-characterized cohort to evaluate the status of the West African epidemic, evaluate the impact of antiretroviral therapy, and investigate factors that impact virologic control and immune recovery in these individuals.

In this study, we established the West African Centre for Cell Biology of Infectious Pathogen (**W**ACCBIP) long-term **H**IV **I**nfection **C**o**h**ort (WHICH Study) made up of newly diagnosed but yet to initiate ART(ART-naïve) and persons already on ART for at least six months (treatment-experienced) living with HIV/AIDS in Ghana, a West African country. This cohort was established in Ghana to include all distinct subgroups of people living with HIV/AIDS, those with HIV-1 and HIV-2 mono- and HIV-1&2 dual infections, and to combine treatment-naïve and experienced participants. It is one of the few of its kind in Africa due to the diversity of the localized epidemic. The WHICH Study will allow a detailed understanding of the dynamics of the Ghanaian epidemic, provide a well-characterized cohort for clinical studies and provide patient samples for detailed mechanistic analysis of complex interactions driving and/or limiting the size of the West African epidemic. The aim of this study was to assess viral load dynamics, immune responses, and organ-level metabolic changes following ART initiation, using interim data from the cohort to provide insights into the evolving HIV epidemic in the Ghanaian context.

This cohort allows for effective targeting of the West African epidemic and provides vital knowledge that will reduce transmission and edge us closer to ending AIDS by 2030.

## Methods

### Study sites and study design

From July 2022 to July 2024 participants were recruited from six health facilities across the country: Greater Accra Regional Hospital (GARH), Tema General Hospital (TGH), Ga West Municipal Hospital, Korle-Bu Teaching Hospital and University Hospital-Legon (UH-L) in the Greater Accra Region and Ho Municipal Hospital in the Volta Region. All selected study sites are located within urban communities. The Greater Accra Region has the highest adult HIV prevalence of 2.47%, whereas the Volta Region has a prevalence of 1.28% [8]; both regions are within the southern part of Ghana. Individuals who were confirmed HIV positive but were yet to initiate ART were classified as ART-naïve while individuals who had been on ART for at least six months were considered ART-experienced. This study used a mixed approach where blood samples, medical records and demographic data for the ART-naïve group were collected at baseline and at six months post-ART initiation. The ART-experienced group was assessed at a single time point for cross-sectional analysis. A purposive sampling approach was used for both the longitudinal and cross-sectional study arms.

Trained nurses, responsible for counselling and ART initiation, recruited participants after the study protocol was explained to them in their preferred language and their written informed consent was obtained. The clinical care plan for the participants was not interrupted during this research. All ART-naïve participants were put on fixed-dose combinations (FDCs) of tenofovir (TDF) + lamivudine (3TC) or emtricitabine (FTC) + dolutegravir (DTG) as per the recommended guidelines for first-line ART in Ghana(9). Medical records were reviewed for information on any known chronic disease conditions, ART regimens for ART-experienced participants and any other current medication. A structured questionnaire was used to collect participants’ demographics, smoking history and other relevant clinical information at baseline and six-month follow-up for ART-naïve and at time of recruitment for the ART-experienced group. From the blood collected at each timepoint, CD4 count, CD8 count, viral load, haemoglobin, aspartate aminotransferase (AST), alanine aminotransferase (ALT) and creatinine were measured for this study and shared with clinical team when results became available. CD4 counts are not routinely measured for routine clinical care and viral loads were only measured six-months post ART in routine clinical care for people living with HIV (PLWH) in Ghana [9].

### Inclusion and exclusion criteria

Adult participants provided informed written consent, whereas children were at least 10 years old provided assent and parental consent. Pregnant women, children younger than 10 years and adults who did not provide informed consent were excluded.

### HIV status verification and STI coinfection testing

HIV screening results received from the health facilities were validated using a World Health Organization (WHO) validated rapid diagnostic kit, SD BIOLINE HIV-1/2 3.0 (Standard Diagnostic Inc., Korea) [10]. Rapid diagnostic tests were used to screen for sexually transmitted infections, including hepatitis B (Wondfo Rapid One Step HBsAg Test Kit), hepatitis C (Wondfo Rapid One Step HCV Test Kit) and syphilis (One Step Strip Style ANTI-TP (Intec Products, INC. (XIAMEN, P. R. China)), following the manufacturer’s instructions and as described elsewhere [11].

### Biochemical and haematological assessments

Biochemical and haematological assessments were conducted on blood samples collected at baseline and at six-month post ART for the ART-naïve group and from the single sample collected from the ART-experienced at recruitment. Biochemical assessments were performed using the Mindray BS-240 chemistry analyser (Shenzhen Mindray Bio-Medical Electronics Co., Ltd., China). Specifically, Alanine aminotransferase (ALT) and aspartate aminotransferase (AST) were used as biomarkers to assess liver damage (Lala et al., 2023), whereas the blood creatinine concentration was measured as a biomarker for kidney damage (Gounden et al., 2024). The Chronic Kidney Disease Epidemiology Collaboration (CKD-EPI 2021) equation was used to estimate glomerular filtration rate (eGFR) as a test for kidney function [12]. This allowed evaluation of disease or therapeutic effects on kidney function and the ability to excrete drug metabolites. A Diatron Abacus 5 haematology analyser (Diatron MI ZRT, Hungary) was used to estimate haemoglobin (Hb) concentration as a marker for anaemia among the participants using K_2_EDTA whole blood samples (Graham et al., 2023).

### CD4+/CD8 + T-cell count and viral load quantification

**A** BD FACSCount™ analyser (BD Biosciences) was used to estimate the CD4 + and CD8 + T lymphocyte counts from EDTA whole blood samples following the manufacturer’s instructions and as previously described [13].

Viral RNA was extracted from plasma using the Quick-RNA Viral Kits (Zymo Research), and HIV-1 viral load was estimated on the QuantStudio™ 5 Real-Time PCR System for Human Identification (Applied Biosystems) using the Bosphore^®^ HIV-1 Quantification Kits (Anatolia Geneworks) following the manufacturers’ instructions. Viral loads obtained in IU/ml were converted to copies/ml by converting 1 IU to 0.7 copies/ml, as indicated by the kit manufacturer, to be comparable with the WHO International Standard for HIV RNA NAT assays (NBSIC code 97/650).

### Definition of terms

Participants were classified to have low education if their highest education was primary school, medium if participants had junior or senior high school education and post-secondary education was considered high. CD4 counts greater than 500 was considered immune recovery, CD4/CD8 ratio greater than one was considered normal while a ratio less than one was considered low. In this study, viral load categories were defined as follows: undetected—viral load below the analytical detection limit of 35 copies/mL; virologic control—viral load between 35 and 1,000 copies/mL; and uncontrolled viremia—viral load greater than 1,000 copies/mL. We defined organ-level metabolic recovery as the restoration of normal kidney function (measured by eGFR), normalization of liver enzyme levels (ALT and AST), and attainment of normal haemoglobin concentration. Kidney function was classified as normal for eGFR greater than 90 mL/min/1.73m2, mild loss of function for eGFR 60 to 89 mL/min/1.73m^2^, moderate loss of function for eGFR of 59 to 30 mL/min/1.73m^2^, severe for eGFR of 15 to 29 mL/min/1.73m^2^ and kidney failure for eGFR less than 15 mL/min/1.73m^2^ [12].

### Data processing and statistical analysis

The raw data were entered into the Survey Solution suite [14] and subsequently analysed using GraphPad Prism (version 8.0.1; San Diego, CA, USA). Descriptive statistics for cohort characteristics are presented as percentages. The laboratory data for ALT, AST, haemoglobin, CD4 count, CD8 count, and viral load are presented as medians (interquartile ranges). The two-tailed Mann‒Whitney U test was used to compare differences between ART-naïve (M0) and 6-month follow-up (M6), M0 and ART-experienced (E0) as well as between M6 and E0, whereas the Wilcoxon matched-pairs signed rank test was used to compare M0 and M6 for matched pairs. The chi-square and where indicated Fisher’s exact test were used to compare the frequencies and assess the associations between CD4 count as well as viral load and various demographic variables as well as laboratory data for M0 and E0. Univariate and multivariate logistic regression analyses were used to assess the risk factors associated with CD4 count and viral load. In some instances, data analysis was conducted with incomplete datasets for certain variables such as viral load, AST, ALT, haemoglobin concentration and creatinine due to missed follow-up visits among ART-naïve participants, compromised sample quality, and logistical challenges during laboratory processing and testing. A *p value* < 0.05 was considered significant in all analyses.

## Results

### Participants’ characteristics

A total of 426 participants were recruited into the WHICH study cohort (Table 1). This consisted of 159 ART-naïve, 267 ART-experienced participants at recruitment and 54 participants at six-month follow. The mean age of the entire cohort was 41.5 years, with the ART-naïve group (37.0 ± 12.0) being significantly younger than the ART-experienced group (44.1 ± 11.9) (*p* < 0.0001). The majority of participants were female (71.6%), with most self-identifying as heterosexual (93.2%), while 17(4.5%) identified as men who have sex with men (MSM) and 6(1.6%) as bisexual. At least 14.3% reported one chronic disease, with hypertension being the most reported 35 (8.2%). Within the cohort, 42 (9.9%) participants were taking herbal medication on a regular basis, while 179 (42.0%) were also taking at least one dietary supplement. The majority had no history of smoking 395 (92.7%). Most participants (97.9%) in the entire cohort were on dolutegravir-anchored therapy, with all ART-naive (100%) being put on the fixed drug regimen of TDF/3TC/DTG (TLD™) combination. Among the ART-experienced group, 60.3% had been on ART for more than five years. Within this cohort, HIV-1 predominated, accounting for 78.6%, whereas HIV-2 accounted for 2.1%, with 19.2% of participants harbouring dual HIV-1/2 infection.

**Table 1:** Cohort characteristics

Characteristics	Number (%)	Characteristics	Number (%)
**Gender**		**ART Regimen**	
Female	305(71.6%)	Dolutegravir-based	417(97.9%)
Male	121(28.4%)	Other regimen	9(2.1%)
**Age groups**			
< 14	3(0.7%)	**Duration on ART(ART-experienced)**	
15–49	307(72.1%)	0–6months	8(3.8%)
50+	116(27.2%)	7–12 months	5(2.2%)
**Educational level**		1–2 years	37(13.9%)
Low	90(21.1%)	3–5 years	51(19.1%)
Medium	250(58.7)	greater than 5 years	161(60.3%)
High	86(20.2%)		
**Marital status**		**Self-Reported Chronic Disease**	
Single/Never	154(36.2%)	Yes	61(14.3%)
Married/Cohabiting	159(37.3%)	No	365(85.7%)
Divorced	61(14.3%)	**Reported chronic disease**	
Widowed	52(12.2%)	Diabetes	11(2.6%)
**Employment status**		Hypertension	35(8.2%)
Unemployed	51(12.0%)	Asthma	3(0.7%)
Student	29(6.8%)	Liver disease	2(0.5%)
Informal	290(68.1)	Renal disease	1(0.2%)
Formal	56(13.1%)	Haematological disease	1(0.2%)
**Possible transmission route**		Tuberculosis	4(0.9%)
Heterosexual	399(93.7%)	Other	4(0.9%)
MSM	20(4.7%)	No/unknown	365(85.7%)
Bisexual	7(1.6%)	**Future participation in CT**	
**Dietary Supplement**		Yes	332(77.9%)
Yes	179(42.0%)	No	94(22.1%)
No	247(58.0%)	**Reasons for Participation (*n* = 322**)	
**Herbal Medication use**		Advancing medical knowledge	84(19.7%)
Yes	42(9.9%)	Financial compensation	8(1.9%)
No	384(90.1%)	Receiving best medical care	72(16.9%)
**Smoking History**		Learn about new treatment	118(27.7%)
No	395(92.7%)	Others may benefit	22(5.2%)
Yes	31(7.3%)	Helping in developing new medication	28(6.6%)

### Sexually transmitted coinfections

Few coinfections are observed with three sexually transmitted diseases that often coinfect with HIV and often complicate disease prognosis and treatment outcomes: HBV, HCV and syphilis [15, 16]. The prevalence of coinfection within the cohort was 9.5% for HBV, followed by 2.6% for syphilis and 0.5% for HCV.

### Biochemical and haematological assessment

The median estimate glomerular filtration rate (eGFR) increased from 96.0 to 113.0 ml/min/1.73 m² in ART-naïve participants over six months post-ART indicating improved kidney function, whereas the ART-experienced group had a median eGFR of 86.0 ml/min/1.73 m². A normal eGFR was more common in ART-naïve (40.5%) than in ART-experienced (24.2%) participants, with low prevalence of end-stage renal failure in both groups. Elevated AST was detected in 15.1% of ART-naïve individuals and 16.7% of ART-experienced individuals, whereas elevated ALT was detected in 5.7% and 2.5%, respectively (Table 2).

**Table 2:** Biomarker of disease progression

Parameter	ART-Naïve	6-Month Follow-up	ART-Experienced	*p*-value
**Kidney Function (eGFR)**				**< 0.0001**
Median (IQR)	96.0 (77.3–115.3)	113.0 (100.0–121.5)	86.0 (67.0–106.0)	
Normal (> 90.0)	62 (40.5%)	44 (84.6%)	55 (24.2%)	
Mild CKD (60–89)	35 (22.9%)	4 (7.7%)	43 (18.9%)	
Moderate CKD (30–59)	5 (3.3%)	4 (7.7%)	17 (7.5%)	
Severe CKD (15–29)	1 (0.7%)	0 (0.0%)	0 (0.0%)	
End-stage CKD (< 15.0)	1 (0.7%)	0 (0.0%)	1 (0.4%)	
Data Unavailable	49 (32.0%)	2 (3.8%)	111 (48.9%)	
**AST Levels (U/L)**				0.2324
Median (IQR)	29.9 (22.5–38.9)	26.5 (19.1–42.1)	26.6 (20.1–36.8)	
Normal AST (8–48)	89 (84.0%)	44 (84.6%)	100 (83.3%)	
Elevated AST (> 48.0)	16 (15.1%)	8 (15.4%)	20 (16.7%)	
Low AST (< 8.0)	1 (0.9%)	0 (0.0%)	0 (0.0%)	
**ALT Levels (U/L)**	14.9 (11.1–22.5)	18.4 (12.7–27.4)	15.6 (11.0–23.1)	0.4757
Normal ALT (7–55)	94 (88.7%)	48 (92.3%)	108 (90.0%)	
Elevated ALT (> 55.0)	6 (5.7%)	4 (7.7%)	3 (2.5%)	
Low ALT (< 7.0)	6 (5.7%)	2 (3.8%)	9 (7.5%)	
**Haemoglobin (g/dL)**				**0.0139**
Median (IQR)	11.9 (10.1–13.0)	12.8 (11.6–13.9)	12.0 (11.2–13.2)	
Anaemia (F:<12.0, M:< 13.0)	90 (61.6%)	23 (44.2%)	55 (46.6%)	
Normal (F:>12.0, M: >13.0)	56 (38.4%)	29 (55.8%)	63 (53.4%)	

Chronic renal failure according to Estimated Glomerular Filtration Rate (CDK-EPI) in ml/min/1,73 m^2^, N (%), IQR: interquartile range, CKD: chronic kidney disease, AST: aspartate aminotransferase, ALT: alanine aminotransferase. F: Female, M: Males

Haemoglobin (Hb) concentrations were measured to assess anaemia burden. In the ART-naïve group, the median Hb level increased from 11.85 g/dL at baseline to 12.80 g/dL after six months, whereas the ART-experienced group had a median Hb level of 12.0 g/dL. Anaemia prevalence was 61.6% in the ART-naïve group and 46.6% in the ART-experienced group at recruitment (Table 2).

### Viral load changes pre and post antiretroviral therapy

**Fig. 1:**
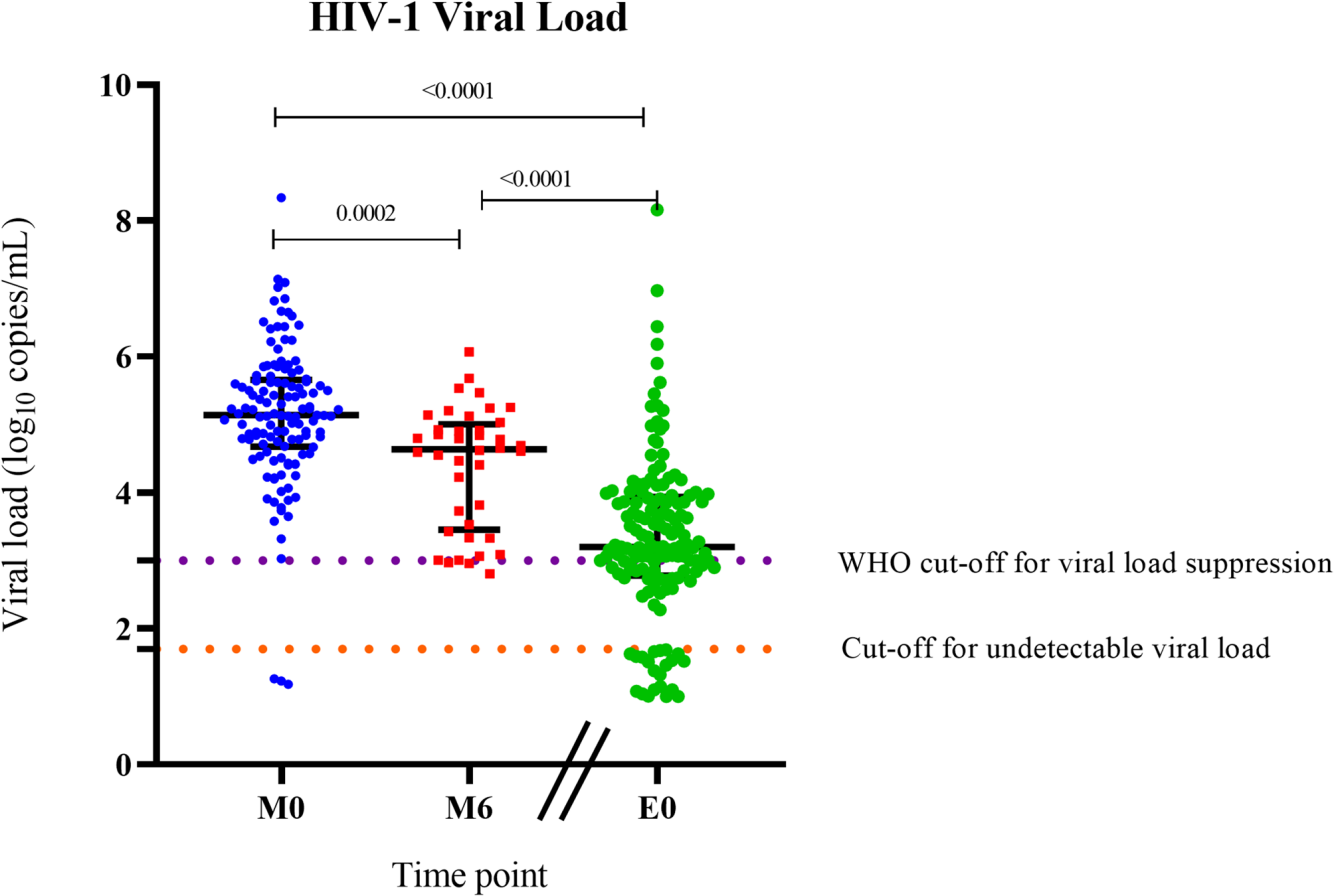
HIV viral load (VL) variation across the cohort. Measured for baseline ART-naïve participants (M0), six months after antiretroviral therapy initiation (M6) and treatment-experienced participants (E0). Thick black lines across each group represent the median VL, violet dotted line set at 1000copies/mL (10^3^) and orange dotted line set to 35 copies/mL. Statistical comparisons were conducted with the two-tailed Mann-Whitney U test

The baseline median viral load in the ART-naïve group was 136,502 copies/ml (log_10_ 5.140). Surprisingly, this number was reduced by only one log to 43,319 copies/ml (log_10_ 4.635) after 6 months of mostly DTG-anchored ART (*p =* 0.0003) (Fig. 1). In the ART-experienced group, the median viral load was 1568 copies/ml (log_10_ 3.20). At recruitment, an undetectable viral load was observed in 3.2% of the ART-naïve participants and in 6.5% of the ART-experienced participants. Virologic control (≤ 1000 copies/mL) was achieved in 25.9% of the ART-experienced individuals, whereas 67.6% had uncontrolled viremia (Table 3) within our cohort.

**Table 3:** HIV treatment outcomes

Recruitment Group	Undetected	Virologic control	Uncontrolled viremia
ART-Naïve n (125)	3(2.4%)	0(0.0%)	122(97.6%)
6-Month follow-up n (41)	1(2.4%)	3(7.3%)	37(90.2%)
ART-Experienced n (149)	7(4.7%)	44(25.5%)	98(65.8%)

Undetected: viral load below the analytical detection limit of the kit (35 copies/ml); virologic control: viral load 35–1000 copies/ml; and uncontrolled viremia: viral load greater than 1000 copies/ml

### T-cell response before and after antiretroviral therapy

**Fig. 2:**
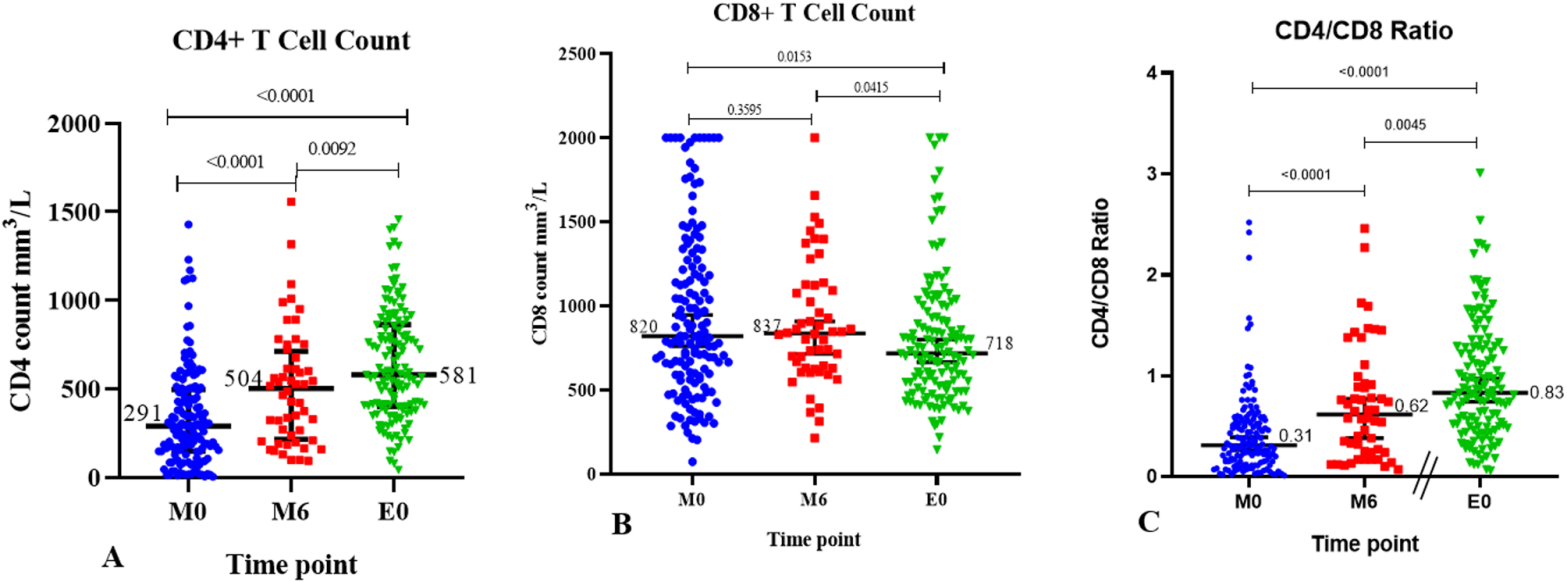
Relative T-cell proportions in whole blood of study participants. (**A**) CD4⁺ T cell count, (**B**) CD8⁺ T cell count, and (**C**) CD4/CD8 ratio. Thick black lines across each group represent the median value. Measurement done for ART-naïve individuals at baseline (M0), six months after therapy initiation (M6), and for baseline treatment-experienced individuals (E0). Statistical comparisons were conducted with the two-tailed Mann-Whitney U test

The median CD4 + T cell counts in the ART-naïve group increased significantly from 291.0 to 504.0 cells/mm³ after six months of ART (*p* < 0.0001), while the ART-experienced group had a higher median count of 581.0 cells/mm³, which was significantly greater than the ART-naïve group at follow-up (*p* = 0.0092). (Fig. 2a). In the ART-naïve group, no statistically significant difference was observed between the median CD8 + T-cell count at baseline and 6 months post ART, but the CD8 + T-cell count was significantly lower (*p* = 0.0153) in the ART-experienced group (718.0 cells/mm^3^) than in the baseline ART-naïve group (820.0 cells/mm^3^) (Fig. 2b). The median CD4+/CD8 + T-cell ratio significantly increased from 0.31 at baseline to 0.62 at 6 months post ART. It was also significantly greater in the ART-experienced group (0.830) than in the six-month follow up in the ART-naïve group (*p* = 0.0045) (Fig. 2c).

### Shifts in HIV staging according to WHO classification based on CD4 + T cell count

In the ART-naïve group, most participants (36.8%) were classified as WHO stage 4 based on CD4 count at baseline. In contrast, 62.0% of ART-experienced individuals were categorized as WHO stage 1 at the time of recruitment (Table 4).

**Table 4:** WHO classification of HIV disease by CD4 + T-cell count

WHO Classification (Cells/mm^3^)	ART-Naïve (*n* = 152)	6-months Post ART (*n* = 54)	ART-Experienced (*n* = 137)
Stage 1 (500–1500)	36(23.7%)	26(49.0%)	85(62.0%)
Stage 2 (350 to 499)	21(13.8%)	7(13.2%)	28(20.4%)
Stage 3/AHD (200 to 349)	39(25.7%)	10(18.9%)	18 (13.1%)
Stage 4/AIDS (< 200)	56(36.8%)	10(18.9%)	6(4.4%)

AHD: Advanced HIV disease, AIDS: acquired immunodeficiency syndrome

### Slow viral load decline despite immune recovery

**Fig. 3:**
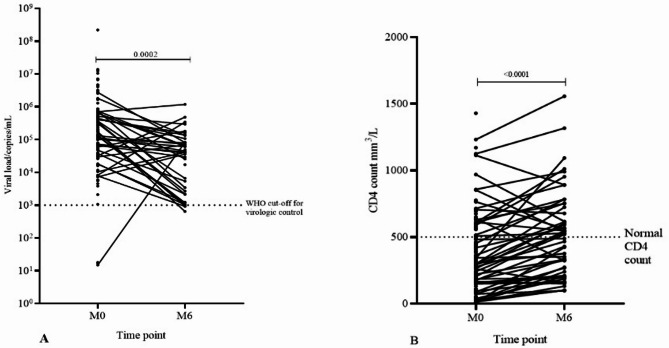
Slow viral load decline despite immune recovery. (**A**) viral load and (**B**) CD4 T-cell count from ART-naïve baseline (M0) to six months (M6) follow up in matched paired samples. CD4 count above 500 cells/mm^3^ were considered normal. Statistical comparison between M0 and M6 was done with Wilcoxon matched pairs signed rank test

The median viral load decreased marginally after 6 months of ART, although the majority remained virally non-suppressed in matched paired samples among ART-naïve group (Fig. 3a). During the same follow-up period, 28.6% of ART-naïve individuals with initially low CD4 counts achieved immune recovery (CD4 count greater than 500 cells/mm^3^) (Fig. 3b). Overall, 49% of participants in the M0 group demonstrated immune recovery by month six (M6).

### Factors associated with uncontrolled viremia

We analysed factors associated with high viral load using Chi-square/Fisher’s exact tests and logistic regression. Univariate analysis revealed that ART status, herbal medication use, CD4 + T-cell count, and HIV type were significant risk factors (Table 5). Multivariate analysis confirmed ART status as an independent predictor, with ART-naïve participants having a significantly greater likelihood of uncontrolled viremia (aOR: 19.49; 95% CI: 4.74–80.14; *p* < 0.0001). Other demographic, clinical, and behavioural factors showed no significant associations.

**Table 5:** Factors associated with uncontrolled viremia

Variables	Descriptive analysis			Logistics Regression Analysis			
	Chi-Square			Univariate		Multivariate	
	**Virologic Control n (%)**	**Uncontrolled viremia n (%)**	**P value**	**OR (95% CI)**	**P value**	**aOR (95% CI)**	**P value**
**Age (years)**							
14–49	33 (63.5)	166 (75.1)	0.089	1.74 (0.92–3.30)	0.091		
> 50	19 (36.5)	55 (24.9)		1			
**Gender**							
Male	16 (30.8)	70 (31.7)	0.8994	1			
Female	36 (69.2)	151 (68.3)		0.96 (0.50–1.84)	0.899		
**ART Status**							
Naïve	3 (5.8)	122 (55.2)	**< 0.0001** ^**$**^	20.13 (6.09–66.53)	**< 0.0001**	19.49 (4.74–80.14)	**< 0.0001**
Experienced	49 (94.2)	99 (44.8)		1		1	
**Education level**							
Low	8 (15.4)	50 (22.6)	0.5165	1.60 (0.69–3.71)	0.274		
Medium	32 (61.5)	125 (56.6)		1			
High	12 (23.1)	46 (20.8)		0.98 (0.47–2.07)	0.96		
**Employment Status**							
Unemployed	6 (11.5)	24 (10.9)	0.4869	1			
Formal	5 (9.6)	41 (18.6)		2.05 (0.57–7.44)	0.275		
Informal	37(71.2)	142 (64.3)		0.96 (0.37–2.52)	0.933		
Student	4 (7.7)	14 (6.6)		0.88 (0.21–3.64)	0.854		
**Possible transmission route**							
Heterosexual	49 (94.2)	206 (93.2)	> 0.9999^$^	1			
Homosexual/Bisexual	3 (5.8)	15 (6.8)		1.19 (0.33–4.27)	0.79		
**Smoking**							
No	50 (96.2)	200 (90.5)	0.2687^$^	1			
Yes	2 (3.8)	21 (9.5)		2.63 (060–11.57)	0.202		
**Self-reported Chronic Disease**							
No	43 (82.7)	186 (84.2)	0.7953	1			
Yes	9 (17.3)	35 (15.8)		0.90 (0.40–2.01)	0.795		
**Herbal medication use**							
No	2 (3.8)	36 (16.3)	**0.0237** ^**$**^	1		1	
Yes	50 (96.2)	185 (83.7)		4.87 (1.13–20.90)	**0.033**	0.64 (0.10–3.96)	0.632
**Dietary supplement**							
No	24 (46.2)	124 (56.1)		1			
Yes	28 (53.8)	97 (43.9)	0.1949	0.67 (0.37–1.23)	0.196		
**CD4 + Count**(***n* = 229**)							
Normal	22(61.1)	74 (38.3)	**0.011**	0.40 (0.19–0.82)	**0.013**	0.80 (0.35–1.83)	0.602
Low	14 (38.9)	119 (61.7)		1		1	
**CD8 + Count** (***n* = 229**)							
Normal	36 (100.0)	191 (99.0)	0.5396^$^		NA		
Low	0(0.0	2 (1.0)			NA		
**CD4/CD8 Ratio (229)**							
Normal	12 (33.3)	36 (18.7)	**0.047**	1			
Low	24 (66.7)	157 (81.3)		2.18 (1.00–4.77)	0.051		
**Haemoglobin** (*n*** = 216**)							
Non-anaemic	17(58.6)	83(44.4)	0.1526	1			
Anaemic	12(41.4)	104 (55.6)		1.78 (0.80–3.92)	0.156		
**eGFR (** ***n*** ** = 201)**							
Normal	16(48.5)	102(60.7)	0.1921	1			
Low	17(51.5)	66(39.3)		0.61 (0.29–1.29)	0.195		
**AST (** *n* ** = 206)**							
Normal	32 (94.1)	143 (83.1)	0.1019	1			
Elevated	2 (5.9)	29 (16.9)		3.25 (0.74–14.30)	0.12		
**ALT (** ***n*** ** = 206)**							
Normal	34(100)	163(94.8)	0.3608^$^		NA		
Elevated	0(0.0)	9(5.2)			NA		
**HIV types**							
HIV-1	34(65.4)	193(87.3)	**0.0007**	1		1	
HIV 1&2	14(26.9)	23(10.4)		0.29 (0.14–0.62)	**0.001**	0.30 (0.06–1.61)	0.16
HIV-2	4(7.7)	5(2.3)		0.22 (0.06–0.86)	0.03	0.25 (0.32–1.86)	0.174
**STI-co-infection**							
No	44(84.6)	199(90.0)	0.26	1			
Yes	8(15.4)	22(10.0)		0.61 (0.25–1.46)	0.264		

ART: Antiretroviral therapy, STI: sexually transmitted infection, eGFR: estimated glomerular filtration rate, AST: aspartate aminotransferase, ALT: alanine aminotransferase; OR: odds ratio, aOR: adjusted odds ratio, NA: not applicable or unable to estimate, $: where indicated Fisher’s exact test are used to analyse data

### Factors associated with immune recovery

With the caveat that small sample sizes may create some bias, univariate logistic regression identified female gender, dietary supplement use, virologic control, low eGFR, and normal AST as factors associated with immune recovery, whereas ART-naïve status, formal employment, a low CD4/CD8 ratio, and anaemia were linked to lower odds of a normal CD4 + count (Table 6). After controlling for potential confounding factors in a multivariate logistic regression model, participants who were ART-naïve had significantly lower odds of having a normal CD4 + count (aOR = 0.35, 95% CI: 0.140–0.85, *p* = 0.021). Similarly, a low CD4/CD8 ratio was inversely associated with normal CD4 + count (aOR = 0.06, 95% CI: 0.02–0.20; *p* < 0.001), and anaemia was also inversely associated with normal CD4 + count (aOR = 0.32, 95% CI: 0.15–0.69; *p* = 0.003). Other variables including gender, renal function (eGFR), AST levels, virologic control, occupation, and dietary status did not show statistically significant associations with normal CD4 count after adjusting for confounders.

**Table 6:** Factors associated with immune recovery (normal CD4 count)

Variables	Descriptive analysis			Logistic Regression Analysis			
	Chi-Square			Univariate		Multivariate	
	**Low CD4** **n (%)**	**Normal CD4** **n (%)**	***P value***	**OR (95% CI)**	***p value***	**aOR (95% CI)**	***p value***
**Age (years)**							
14–49	134(79.8)	92(71.3)	0.0908	1	0.092		
> 50	34(20.2)	37(28.7)		0.63 (0.37–1.08)			
**Gender**							
Male	72(42.9)	23(17.8)	< 0.0001	1		1	
Female	96(57.1)	106(82.2)		3.46 (2.01–5.96)	**< 0.0001**	2.03 (0.89–4.63)	0.091
**ART Status**							
Naïve	116(69.0)	38(29.5)	< 0.0001	0.19 (0.11–0.31)	**< 0.0001**	0.35 (0.14–0.85)	**0.021**
Experienced	52(31.0)	91(70.5)		1		1	
**Education level**							
Low	35(20.8)	33(25.6)	0.2426	1.17 (0.67–2.06)	0.587		
Medium	93(55.4)	75(58.1)		1			
High	40(23.8)	21(16.3)		0.65 (0.35–1.20)	0.167		
**Employment Status**							
Unemployed	17(10.1)	21(16.3)	0.1396	1		1	
Informal	110(65.5)	87(67.4)		0.64 (0.32–1.29)	0.211	1.22 (0.38–3.91)	0.744
Formal	27(16.1)	11(8.5)		0.33 (0.13–0.85)	**0.022**	0.49 (0.11–2.24)	0.354
Student	14(8.3)	10(7.8)		0.58 (0.21–1.63)	0.299	0.75 (0.14–4.18)	0.745
**Possible transmission route**							
Heterosexual	151(89.9)	123(95.4)	0.1237^$^	1			
Homosexual/Bisexual	17(10.1)	6(4.6)		0.43 (0.17–1.13)	0.088		
**Smoking**							
No	149(88.7)	121(93.8)	0.1291	1			
Yes	19(11.3)	8(6.2)		0.52 (0.22–1.23)	0.134		
**Self-reported Chronic Disease**							
No	141(83.9)	114(88.4)	0.276	1			
Yes	27(16.1)	15(11.6)		0.69 (0.35–1.35)	0.278		
**Herbal medication use**							
No	25(14.9)	15(11.6)	0.4156	1			
Yes	143(85.1)	114(88.4)		0.75 (0.38–1.49)	0.417		
**Dietary supplement**							
No	87(51.8)	49(38.0)		1		1	
Yes	81(48.2)	80(62.0)	0.018	1.75 (1.10–2.89)	**0.018**	0.76 (0.34–1.72)	0.531
**Viral load (** ***n*** ** = 233)**							
Virologic control	14(10.5)	22(22.0)	0.0165	1		1	
Uncontrolled viremia	119(89.5)	78(78.0)		2.53 (1.22–5.25)	**0.013**	0.80 (0.28–2.33)	0.686
**CD8 + Count (** ***n*** ** = 289)**							
Normal	166(98.8)	121(100)	0.5115^$^	NA			
Low	2(1.2)	0(0.0)		NA			
**CD4/CD8 Ratio (289)**							
Normal	7(4.2)	55(45.5)	< 0.0001	1		1	
Low	161(95.8)	66(54.5)		0.05 (0.02–0.12)	**< 0.0001**	0.06 (0.02–0.20)	**< 0.0001**
**Haemoglobin (** ***n*** ** = 266)**							
Non-anaemic	59(38.8)	62(54.4)	0.0116	1		1	
Anaemic	93(61.2)	52(45.6)		0.55 (0.34–0.90)	**0.018**	0.32 (0.15–0.67)	**0.003**
**eGFR (** ***n*** ** = 248)**							
Normal	95(66.4)	44(41.9)	0.0001	1		1	
Low	48(33.6)	61(58.1)		2.74 (1.63–4.62)	**< 0.0001**	1.92(0.93–4.00)	0.079
**AST (** ***n*** ** = 253)**							
Normal	116(80.0)	100(92.6)	0.0051	1		1	
Elevated	29(20.0)	8(7.4)		3.12 (1.37–7.15)	**0.007**	1.00 (0.36–2.84)	0.994
**ALT (** ***n*** ** = 253)**							
Normal	137(94.5)	106(98.2)	0.1963^$^	1			
Elevated	8(5.5)	2(1.8)		3.10 (0.64–14.88)	0.158		
**HIV types**							
HIV-1	162(96.4)	126(97.7)	0.7363^$^	1			
HIV 1&2 + HIV-2	6(3.6)	3(2.3)		0.77 (0.18–3.29)	0.726		
**STI coinfection**							
No	152(90.5)	113(87.6)	0.7933	1			
Yes	16(9.5)	16(12.4)		1.35 (0.65–2.80)	0.429		

ART: Antiretroviral therapy, STI: sexually transmitted infection, eGFR: estimated glomerular filtration rate, AST: aspartate aminotransferase, ALT: alanine aminotransferase, OR: odds ratio, aOR: adjusted odds ratio, CI: confidence interval, $: where indicated Fisher’s exact test are used to analyse data

## Discussion

Achieving the Joint United Nations Programme on HIV/AIDS (UNAIDS) goal of eliminating HIV transmission by 2030 requires a comprehensive understanding of diverse sub-epidemics. In this context, the WHICH study was designed to provide critical insights into the HIV epidemic in Ghana, serving as a representative model of the broader sub-epidemic in West Africa.

In this study, 71.6% of the participants were female, which is consistent with the sex distribution of HIV infection in Ghana (64%) [8] and elsewhere [17]. Most participants (72.1%) were aged 15–49 years, representing the demographic most affected by HIV and likely to remain on treatment for several decades. This requires robust healthcare systems and sustained access to antiretroviral therapy (ART) to ensure effective management over several decades [18, 19]. Heterosexual contact was the main mode of HIV transmission in the study (93.7%), aligning with patterns seen across Africa [20]. In contrast, HIV transmission in Western countries is more commonly driven by men who have sex with men (MSM) [21]. A small proportion of participants (1.6%) identified as bisexual, a key group in transmission dynamics, as they may act as a bridge for HIV and other STIs between high-risk male-to-male networks and lower-risk female partners [22]. Chronic diseases were reported by 14.3% of the participants, with hypertension being the most common (8.2%), which is consistent with findings in sub-Saharan Africa [23].

We found a prevalence of 9.5% for HBV in our cohort, which is higher than that reported in earlier reports from Ghana, which reported 6.5–7.0% among PLWH [24, 25]. The prevalence of HCV in our study was 0.5%, which was consistent with an earlier report from Ghana which also reported a seroprevalence of 0.57% [26]. This low prevalence of HCV reflects the overall low burden of hepatitis C observed across sub-Saharan Africa compared global report of 2.4% [27] and 5.3% in Thailand [28]. Nearly all the participants were on a DTG-based regimen (97.9%) (Table 1). This reflects the wide adoption of the WHO recommendation for the use of DTG-based regimens as first-line ART [10].

The significant increase in eGFR observed among ART-naïve individuals over the first six months of therapy suggests a potential renal recovery following ART initiation, likely due to reduced HIV-associated renal inflammation [29]. In contrast, ART-experienced individuals exhibited a lower median eGFR (86.0 mL/min/1.73 m²) and a smaller proportion with normal kidney function. This may reflect the cumulative nephrotoxic effects of prolonged ART exposure or age-related decline, as this group was notably older than the ART-naïve cohort [29, 30]. Similarly, haemoglobin concentrations improved in the ART-naïve group during follow-up, indicating a reduction in HIV-related anaemia with ART initiation [31]. The prevalence of elevated liver enzymes (AST and ALT) remained low across both groups, although ART-experienced individuals showed a slightly higher frequency of elevated AST, which may suggest ongoing hepatic stress or long-term ART-related hepatotoxicity [31].

The viral load was significantly reduced in the ART-naïve group six months after initiating ART. However, a substantial proportion ART-naïve of participants (90.2%) had uncontrolled viremia after six months on ART. Approximately 66% of participants in the ART-experienced group had uncontrolled viremia despite the majority (51.5%) having been on treatment for more than five years. This falls short of the third indicator in the 95–95–95% strategy of achieving 95% viral suppression among persons receiving ART by 2030 [32]. In contrast, Gebremedhin, Aynalem [33] reported a 91.7% viral load suppression rate after six months of DTG-based ART in an Ethiopian cohort. Similarly, Hoenigl, Chaillon [34] reported an 88% suppression rate at 48 weeks in a U.S. longitudinal study. The high viral loads observed in this study may reflect late diagnosis, regional circulation of high-replicative HIV-1 subtypes (CRF02_AG), or potential challenges in treatment adherence—factors previously documented in sub-Saharan African populations, or the varying susceptibility of HIV subtypes to ART across regions may [6, 35, 36]. Additionally, unlike participants in the Ethiopian and U.S. studies who initiated ART early in infection, individuals in this cohort began treatment at more advanced disease stages (stages 3 and 4, Table 4). The 3.2% of ART-naïve participants with undetectable viral load may represent the presences of elite controller —individuals who naturally suppress HIV replication to undetectable levels without antiretroviral therapy. A further study of this subset may provide clues to guide functional cure strategies as well as identifying biomarkers that predict better disease outcomes or treatment responses [37].

A significant increase in CD4 + T-cell counts, averaging 212.5 cells/mm³, was observed six months after initiating antiretroviral therapy in the ART-naïve group and this was significantly higher in the ART-experienced group. These findings highlight the importance of sustained ART in restoring immune function and preventing progression to AIDS. HIV-specific CD8 + T cells significantly decrease with prolonged ART, indicating reduced immune activation due to effective viral suppression [38]. This reduction in immune activation is also reflected in the increase in the CD4/CD8 ratio at six months post-ART as well as within the ART-experienced group. Immune reconstitution, which is essential for controlling viral replication and improving health outcomes, has been widely reported in both adults and paediatric patients [33, 39–41].

Interestingly, despite evidence of immune recovery reflected by median CD4 + T-cell counts exceeding 500 cells/mm³ at the six-month follow-up for the ART-naïve and ART-experienced groups; viral loads remained elevated, with only modest reductions observed following six months of antiretroviral therapy in the ART-naïve group. This observation may be attributed to several factors, including compensatory proliferation of CD4 + T cells to counteract immune destruction in the presence of ongoing viral replication as well as increased production of naïve T cells from the bone marrow and thymus, particularly in younger participants [42, 43]. Additionally, partial suppression of viral replication through ART may attenuate the virus’s pathogenicity sufficiently to allow immune recovery, even in the presence of a high viral load [41]. Variation in HIV subtypes or genetic mutations may also contribute to attenuated pathology, limiting immune destruction or impairing cell–cell spread despite high viral loads [6, 44]. Furthermore, drug resistant HIV strains especially among the ART-experienced participants as have been reported in Ghana may contribute to this observation [45, 46]. HIV viral load alone is therefore not enough to assess for ART outcomes but should be multifaceted to include immune monitoring and clinical outcomes.

ART-naïve individuals were independently associated with a greater risk of having uncontrolled viremia, likely aggravated by their advanced disease status at diagnosis (Table 4). This underscores the importance of early detection of HIV and frequent testing for viral load, as a low viral reservoir can increase the ability of ART to fully suppress viremia [47–49]. Interestingly, while herbal medication use, low CD4⁺ counts and HIV types were significant in univariate models, their effects were attenuated in the multivariate context. This suggests that these associations may be confounded by ART exposure, as individuals not receiving ART may be more likely to rely on alternative therapies or present with lower immune reconstitution.

A normal CD4/CD8 ratio was strongly associated with immune recovery, which is consistent with prior studies linking a balanced CD4/CD8 ratio to improved immune responses and better treatment outcomes [48]. The CD4/CD8 ratio has been proposed as a surrogate marker for immune recovery and decreased immune activation [50]. ART-experienced participants had significantly higher odds of having a normal CD4 count compared to ART-naïve individuals. This finding aligns with existing evidence demonstrating the beneficial effect of sustained ART on immune recovery, particularly CD4 T-cell reconstitution [51]. Additionally, the absence of anaemia was significantly associated with normal CD4 levels. Anaemia is a common haematological complication in people living with HIV and has been linked to advanced disease, opportunistic infections, and poor immune recovery [31]. Gender, renal function (eGFR), AST levels, virologic control, occupation, and dietary supplement intake were not independently associated with immune recover (normal CD4 count). This suggests that while these variables may have biological relevance, their effects are not independently predictive of CD4 normalization when considered alongside key immunologic and treatment-related variables.

## Conclusion

This study characterizes a cohort representative of the West African HIV epidemic, providing insights into treatment outcomes in Ghana. DTG-based ART led to immune recovery and reduced immune activation, reflecting improved health, although uncontrolled viremia remained high. This highlights the need for continuous viral load monitoring, adherence counselling, and timely regimen switching. Further studies, including drug resistance testing and whole-genome sequencing, are needed to identify drug resistance mutations. Although the study was limited to a 6-month follow-up with ART-naïve participants, the cohort provides a valuable platform for exploring local HIV dynamics, cure research, and clinical trials. The observed poor virologic control, despite improvements in immune and organ-level metabolic function, highlights the need for ongoing monitoring, particularly as the cohort grows.

## Electronic supplementary material

Below is the link to the electronic supplementary material.

Supplementary Material 1

## Data Availability

No datasets were generated or analysed during the current study.

## Abbreviations

3TC: Lamivudine
AHD: Advanced HIV Disease
AIDS: Acquired immunodeficiency syndrome
ALT: Alanine Aminotransferase
aOR: Adjusted Odds Ratio
ART: Antiretroviral Therapy
AST: Aspartate Aminotransferase
BD: Becton, Dickinson and Company
CAN: Crick African Network
CD4: Cluster of Differentiation 4
CD8: Cluster of Differentiation 8
CI: Confidence Interval
CRF: Circulating Recombinant Form
DNA: Deoxyribonucleic Acid
DTG: Dolutegravir
ECBAS: Ethics Committee for Basic and Applied Sciences
eGFR: Estimated Glomerular Filtration Rate
FDC: Fixed-Dose Combination
FTC: emtricitabine
GHS-ERC: Ghana Health Service Ethics Review Committee
GMS II: Ghana Men’s Study II
Hb: Haemoglobin
HBV: Hepatitis B Virus
HCV: Hepatitis C Virus
HIV: Human Immunodeficiency Virus
HPTN: HIV Prevention Trials Network
IU/ml: International Units per millilitre
M0: Baseline Measurement for ART-Naïve Participants
M6: Measurement at Six Months After ART Initiation
MSM: Men Who Have Sex with Men
NAT: Nucleic Acid Test
NCDs: Noncommunicable Diseases
OR: Odds Ratio
PCR: Polymerase Chain Reaction
PLWH: People Living with HIV
PrEP: Pre-exposure Prophylaxis
RNA: Ribonucleic Acid
SD: Standard Deviation
STI: Sexually Transmitted Infection
TDF: Tenofovir
TLD: Tenofovir/Lamivudine/Dolutegravir
URF: Unique Recombinant Form
VL: Viral Load
WACCBIP: West African Centre for Cell Biology of Infectious Pathogens
WHO: World Health Organization
WHICH Study: WACCBIP Long-Term HIV Infection Cohort
