# Supplementary material for: Slow virologic control but strong immune and metabolic recovery with dolutegravir-anchored therapy in an HIV cohort in Ghana
Source: Virol J. 2025 Jul 19;22:247. doi: 10.1186/s12985-025-02873-w (PMC12275435; doi:10.1186/s12985-025-02873-w)
Supplement: Supplementary file 1 — Supplementary Material 1 [file 12985_2025_2873_MOESM1_ESM.docx]

**Supplementary Methods**

**HIV Testing at ART Clinics**

To determine the sero-status of participants, the testing algorithm adopted in Ghana which is based on WHO recommendations for testing in population with low prevalence of HIV was used (10). This required the use of three different rapid diagnostics test (RDTs) kits. First Response HIV 1&2 (Premier Medical Corporation Private Limited, India) being the first test kit, Oraquick HIV 1&2 (OraSure Technologies, Inc, USA) as the second test kit and SD Bioline HIV 1&2 (Abbott Diagnostics Scarborough, Inc., USA) as the third test for the general population (9, 10). Persons who are positive for all the three RDTs were classified as sero-positive.

**Antiretroviral therapy initiation**

Participants clinical care was not interrupted during this research. All participants were put on Fixed Dose Combinations (FDC) as per recommended guideline for first line ART in Ghana. The first line regimen is made up of Tenofovir (TDF) + Lamivudine (3TC) (or Emtricitabine (FTC)) + Dolutegravir (DTG), where virologic failure is confirmed with viral load, a second line regimen made up of Zidovudine (AZT/ZDV) + Lamivudine (3TC) (or Emtricitabine (FTC)) + Lopinavir/r (LPV/r) (or Atazanavir/r, ATV/r) were used (9). A third line or salvage therapy is available for those who have failed second line treatment which is made up of Darunavir/r (DRV/r) + Raltegravir (RAL) + 1 or 2 NRTI (9).

**Blood sample collection and processing**

Venous blood (ten ml) was collected from each patient and divided into a BD Vacutainer® K_2_EDTA tubes and BD Vacutainer^®^ SST^TM^ tubes (BD Biosciences, UK). K_2_EDTA whole blood aliquot was used for haemoglobin and CD4/CD8 + T cell count. The remaining portion was centrifuged at 2500 rpm for 10 minutes to separate plasma, Peripheral blood mononuclear cells (PBMCs) and Red Blood Cells (RBCs). PBMC isolation was performed using density gradient separation (Lymphoprep^TM^, STEMCELL Technologies) as previously described (56) and stored in a  Nalgene Mr. Frosty™ Freezing Container (VWR International, LLC.) in -80^o^C freezer for 24 hours prior to liquid nitrogen storage as previously described (57). Blood samples collected in serum separator tube were used to obtain serum for downstream analysis.

**Supplementary Table 1. Reference ranges for Biochemical and Haematological assays**

| Parameter | Classification | Range | Reference |
| --- | --- | --- | --- |
| Kidney Function (eGFR) | Normal | > 90 |  |
| mL/min/1.73m² | Mild loss of function | 60–89 | (58) |
|  | Moderate loss of function | 30–59 |  |
|  | Severe loss of function | 15–29 |  |
|  | End stage renal failure | < 15 |  |
| Haemoglobin (g/dL) | Anaemic (females) | < 12.0 | (Chaparro & Suchdev, 2019) |
|  | Anaemic (males) | < 13.0 |  |
|  |  |  |  |
| AST(U/L) | Low | < 8 |  |
|  | Normal | 8 - 48 |  |
|  | Elevated | > 48 | (59) |
|  |  |  |  |
| ALT(U/L) | Low | < 7 |  |
|  | Normal | 7–55 |  |
|  | Elevated | > 55 |  |

eGFR: estimated glomerular filtration rate, AST: aspartate aminotransferase, ALT: alanine aminotransferase
